# Expanding the chaperone code

**DOI:** 10.1016/j.cstres.2026.100219

**Published:** 2026-09-07

**Authors:** Mark R. Woodford, Andrew W. Truman

**Affiliations:** 1 Department of Urology, SUNY Upstate Medical University, Syracuse, NY 13210, USA; 2 Upstate Cancer Center, SUNY Upstate Medical University, Syracuse, NY 13210, USA; 3 Department of Biochemistry & Molecular Biology, SUNY Upstate Medical University, Syracuse, NY 13210, USA; 4 Department of Biological Sciences, The University of North Carolina at Charlotte, Charlotte, NC 28223, USA; 5 Center for Computational Intelligence to Predict Health and Environmental Risks, University of North Carolina at Charlotte, Charlotte, NC, 28223, USA

Molecular chaperones safeguard protein folding and maturation, thereby maintaining cellular homeostasis and promoting survival during stress.^1^, ^2^, ^3^ This classical view continues to expand with descriptions of signaling networks that direct the remarkable tunability and plasticity of chaperone function. The emerging concept of the “Chaperone Code” points to a complex regulatory architecture that includes post-translational modifications (PTMs^a^) layered on protein-protein interactions, localization, complex composition, and cellular context.^4^^,^^5^ This Special Issue highlights the expanding dimensions of this code across a variety of chaperone families and experimental systems. Several comprehensive reviews encapsulate the current state of Chaperone Code research and describe tools for its evaluation. Backe et al provide a broad framework for understanding how a variety of PTMs regulate Hsp70 and Hsp90,^6^ while the Truman lab describes the impact of PTMs on the function of the Hsp70 co-chaperones Ydj1/DNAJA1.^7^ Woodford and colleagues expand on this concept by outlining the nascent understanding of molecular chaperone PTMs in mitochondria, where the unique challenges of protein import and folding create a specialized regulatory environment for the mitochondrial chaperone network.^8^ Guarra et al further outline computational approaches for resolving the complex relationship between Hsp90 conformational dynamics, PTMs, ligands, clients, and co-chaperones, which may ultimately allow prediction of chaperone behavior.^9^ These works highlight that the code is not necessarily written by a single modification but by the combinatorial integration of multiple regulatory signals.

This idea of a regulatory program extends beyond the chaperones themselves. Alasady and Mendillo describe a complementary “heat shock factor code,” in which HSF paralogs, their abundance and localization, PTMs, and protein interactions specify distinct transcriptional programs.^10^ These perspectives suggest that protein folding is governed by interconnected codes operating from transcriptional specification to post-translational control of the chaperone machinery.

Beyond topic reviews, Goutami et al describe altered lysine acetylation of HSPA2 and other chaperones in idiopathic male infertility, connecting the chaperone landscape with oxidative stress and impaired spermatogenesis, and identifying HSPA2 as a potential disease biomarker.^11^ Further, Camara et al illustrate the therapeutic potential of directly manipulating the Chaperone Code. The authors report the development of a high-throughput strategy to identify inhibitors of HYPE-mediated AMPylation of BiP, facilitating therapeutic targeting of enzymes that write the Chaperone Code.^12^

Collectively, these studies reveal that the Chaperone Code is not a static molecular signature but a dynamic language through which cells tune protein folding and activity in response to environmental and physiological demands. Learning how to read, write, or erase this language may provide a new framework for understanding disease and for designing therapies to selectively reprogram chaperone function.

## Footnotes

a PTMs: post-translational modifications
